# Influence of bone anatomical morphology of mandibular molars on dental implant based on CBCT

**DOI:** 10.1186/s12903-021-01888-3

**Published:** 2021-10-15

**Authors:** Zhuo-lin Kong, Ge-ge Wang, Xue-ying Liu, Zhang-yan Ye, Dong-qian Xu, Xi Ding

**Affiliations:** Department of Stomatology, The First Affiliated Hospital of Wenzhou Medical University, Nanbaixiang Ouhai District, Wenzhou, 325000 Zhejiang People’s Republic of China

**Keywords:** Mandibular molar, Alveolar bone morphology, Cone-beam computed tomography, Dental implant

## Abstract

**Background:**

To apply CBCT to investigate the anatomical relationship between the mandibular molar and alveolar bone, aimed to provide clinical guidelines for the design of implant restoration.

**Methods:**

201 CBCT data were reevaluated to measure height of the alveolar process (EF), width of the alveolar process (GH), width of the basal bone (IJ), the angle between the long axis of the first molar and the alveolar bone (∠a) and the angle between the long axis of the alveolar bone and basal bone (∠b). The angle and width were measured to determine the implant-prosthodontic classification of the morphology in the left lower first molar (36) and right lower first molar (46). All measurements were performed on the improved cross-sectional images.

**Results:**

EF, GH and IJ were measured as (10.83 ± 1.31) mm, (13.93 ± 2.00) mm and (12.68 ± 1.96) mm for 36, respectively; and (10.87 ± 1.24) mm, (13.86 ± 1.93) mm and (12.60 ± 1.90) mm for 46, respectively. No statistical significance was observed in EF, GH, IJ, ∠a and ∠b between 36 and 46 (all *P* > 0.05). The morphology was divided into three categories including the straight (68.7–69.2%), oblique (19.9–20.4%) and concave types (11%). Each type was consisted of two subcategories.

**Conclusions:**

The proposed classification could provide evidence for appropriate selection and direction design of the mandibular molar implant in clinical. The concave type was the most difficult to implant with the highest risk of lingual perforation. The implant length, width, direction required more attention.

## Background

The implant position and angulation serve as the basis for ensuring the long-term stability of the implant and for reducing the occurrence of mechanical complications [1–3]. The implant should be aligned with the long axis of the restoration and the inclination should be minimized. The occlusal force should be conducted along the axial direction of the implant to reduce the lateral force. An ideal mandibular molar implant is inserted through the central fossa of the crown and points to the functional cusp of the opposing maxillary tooth (maxillary palatal cusp), suggesting that the long axis of the implant is the same as that of the original natural tooth crown [1, 2].

The mandibular morphology is likely to change due to the congenital factors of the mandible including the mylohyoid line, submandibular fossa, and sublingual fossa, or the acquired factors, such as tooth loss, time after tooth loss, etc.[4–9]. The perforation on the buccal or lingual side may occur multiple complications, such as bleeding, airway obstruction, inflammatory infection, and even pulmonary embolism, mediastinal inflammation, and upper respiratory obstruction in lingual perforation have been reported [1, 10–14]. Therefore, it is of great significance to study the morphology and bone thickness of the buccal-lingual fracture of the posterior teeth of the mandible, aiming to improve the success rate of implantation and reduce the risk of intraoperative complications. Currently, the most commonly used imaging examination method is panoramic radiography, whereas it is limited to two-dimensional images, which lacks buccal and lingual information and has the disadvantages of image distortion and enlargement [5, 10]. Therefore, some stomatologists advocate the application of computed tomography (CT) for preoperative implant planning, whereas traditional CT also has the disadvantages of high cost and large radiation [1, 15]. Based on the above considerations, cone-beam CT (CBCT) was introduced into the field of stomatology, which yields less radiation, accurate images, and rapid scan time [15–17].

Quirynen et al. had applied CT to measure the morphology of the mandible, whereas this study was limited to the inter-foraminal region [10]. Watanabe et al., Herranz-Aparicio et al., and Parnia et al. adopted CT to measure and classify the mandibular morphology [4, 13, 18]. Chan et al*.* and Gallucci et al. began to utilize CBCT to analyze and classify the morphology of the mandibular molar region based on alveolar bone resorption after missing teeth [1, 9]. Nevertheless, the relationship between the long axis of the crown and the morphology of the jawbone is not considered, and the inclusion criteria are not uniform.

In this study, CBCT was employed to investigate investigate the positional relationship between the mandibular molar and the alveolar bone. The mandibular bone was measured at the lateral fault where the long axis of the mandibular first molar was located. The impact of implant orientation was evaluated to provide a reference for the design of implant restoration in the mandibular molar region.

## Methods

### Sample collection

This study was approved by the institutional review boards of the First Affiliated Hospital of Wenzhou Medical University (Issuing Number: R025). This retrospective study evaluated CBCT scans for the positional relationship between the mandibular first molar and the alveolar bone in 201 patients (100 males, 101 females) from June 2018 to April 2020.

All images were obtained from the same CBCT machine (KaVo 3D eXam) with the minimum filtration equivalent of 120 kVp, the scanning time of 4.8 s, the tube current of 3–7 mA, the number of scanning layers of 320 and the thickness of each layer of 0.3 mm. The scans used in the present study were selected from the CBCT database and were not specifically acquired for this publication.

Images selected for this study had to fulfill the following inclusion criteria: (1) those with complete dentition of the right to the left mandibular second molar; (2) no obvious malformation; (3) no defects or abnormalities in the development of the mandibular posterior teeth; (4) the mandibular posterior teeth were basically intact, without serious caries, wedge-shaped defects, wear or abrasion; (5) no alveolar ridges were clearly absorbed; (6) the mandible was free from pathological diseases and deformities; (7) CBCT images were clear and weightless shadow [1, 11, 19].

The exclusion criteria were: (1) those with moderate to severe or progressive periodontitis; (2) abnormal tooth development, such as apical cysts, intra-root resorption and extra-root resorption, etc.; (3) pathological factors; (4) history of dental and jaw trauma; (5) history of orthodontic treatment [1, 11, 19].

### Measurement of plane, long axis of the tooth, alveolar process and basal bone

E-3D Medical Software V16.19 (Central South University, Changsha, China) was utilized to load the CBCT imaging data. The criteria to record the measurement of plane, long axis of the tooth, alveolar process, basal bone and angle measurement method of calculation were referenced from previous studies [9, 20–22].

**Fig. 1:**
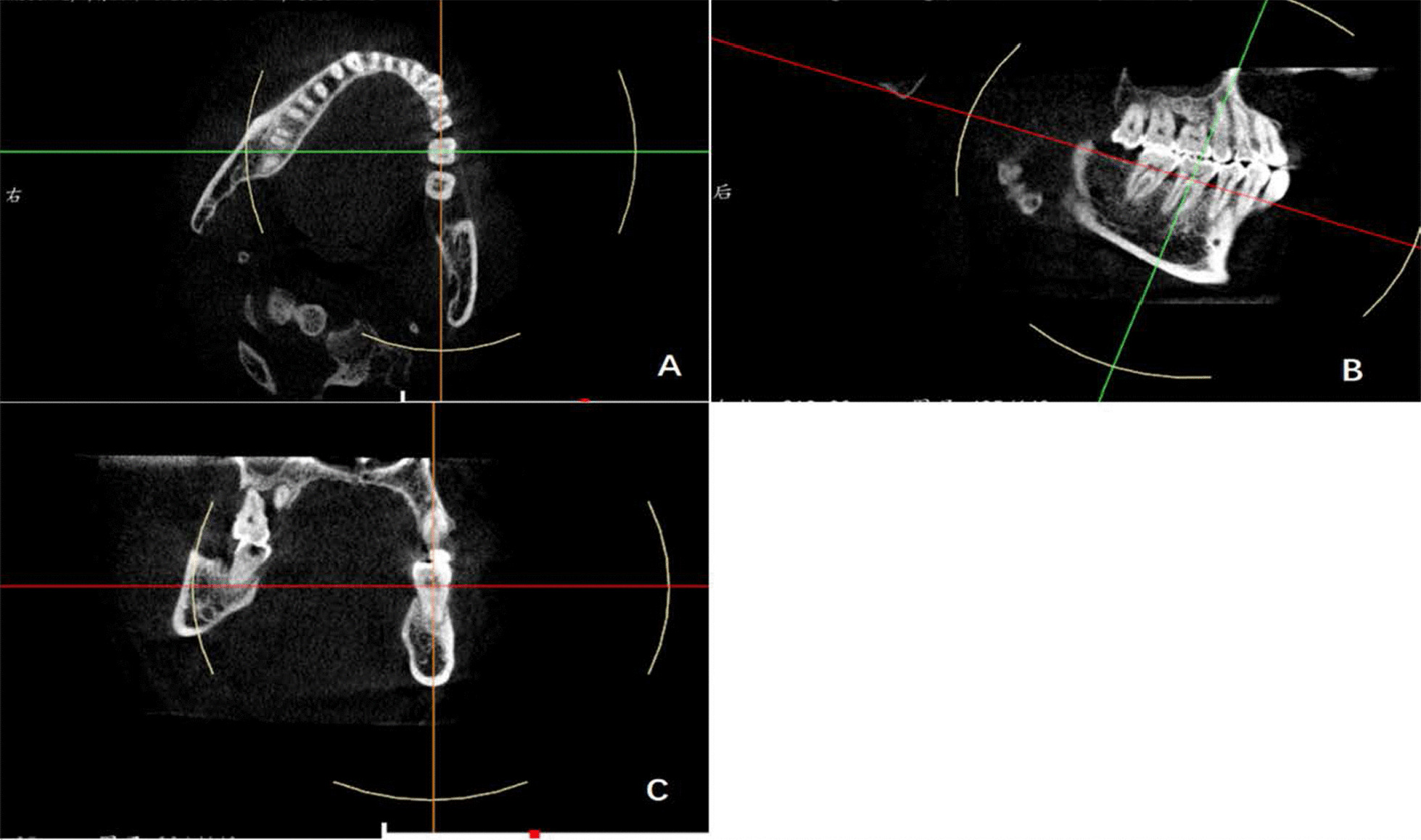
Determination of the measurement plane. **a** The horizontal plane was adjusted to the neck of the lower first molar and the cross-sectional plane passed through the midpoint of the tested tooth mesiodistally. **b** The improved cross-sectional plane aligned along the long axis of the tooth. **c** The selected measurement plane paralleled the improved cross-sectional plane when the plane showed complete root in the single-rooted molar or the mesial and distal root bifurcations displayed complete mesial root

The measurement plane for the tooth position was determined by adjusting the coordinate axis. The details of the adjustments were as follows. (1) The tooth horizontal plane was adjusted to the neck of the tested tooth; (2) the cross-sectional plane passed through the midpoint of the tested tooth mesiodistally; (3) the improved cross-sectional plane aligned along the long axis of the tooth until the plane showed complete root in the single-rooted molar, or the mesial and distal root bifurcations displayed complete mesial root (Fig. 1).

**Fig. 2:**
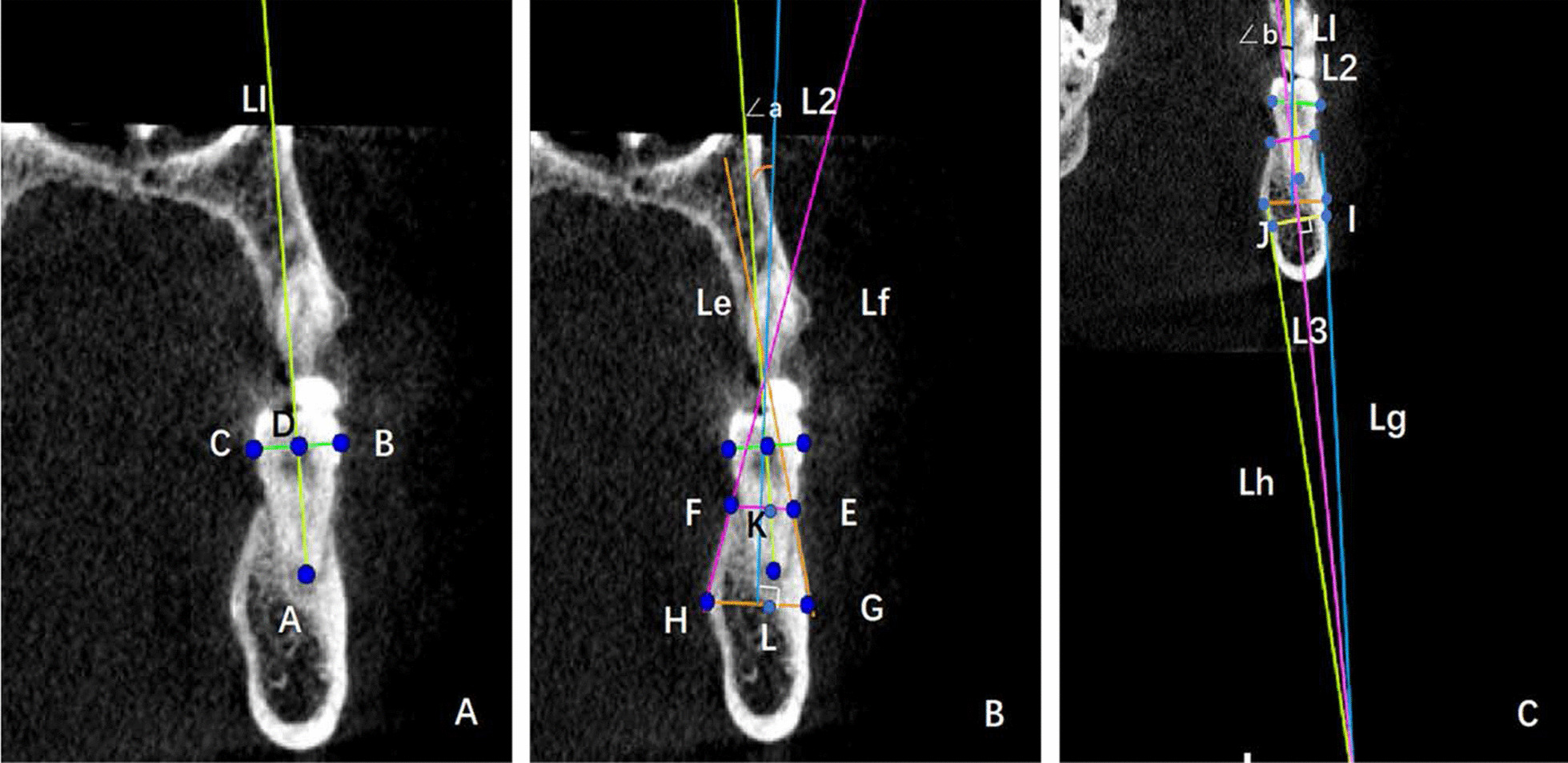
Data measurement. **a** The long axis of the tooth was determined by connecting the apex of the mesial root (point A) and the midpoint of a line drawn from the abrupt point of the buccal counterpart (point B) to the lingual counterpart (point C). **b** The long axis of the alveolar process (line 2) was marked by bisecting the buccal line of the alveolar process (line Le) and lingual line of the alveolar process (line Lf). The upper internal angle **a** was formed by L1 and L2. The width GH was between the buccal and lingual alveolar plates at the lowest point of the alveolar bone and perpendicular to L2. KL was between the midpoint of EF and GH. **c** The long axis of the basal bone (line 3) was marked by bisecting the buccal basal bone surface (line Lg) and lingual basal bone surface (line Lh). The upper internal angle **b** was formed by L2 and L3. The width IJ was between the buccal and lingual basal plates at the highest point of the basal bone and perpendicular to L3.Distance EF was between the buccal and lingual alveolar crest

The long axis of the tooth was determined by connecting the apex of the mesial root (point A) and the midpoint of a line drawn from the abrupt point of the buccal crown surface (point B) to the lingual counterpart (point C) (Fig. 2a).

#### The long axis of the alveolar process

On the selected measurement plane, both the buccal line (line Le) and the lingual line (line Lf) were marked by a line best-fit to the buccal alveolar surface and the lingual alveolar surface, respectively. The alveolar line (line L2) was marked by bisecting the buccal and lingual lines, which indicated the angulation of the alveolar process on the selected measurement plane (Fig. 2b).

#### The long axis of the basal bone

On the selected measurement plane, both the buccal line (Lg) and the lingual line (Lh) were marked by a line best-fit to the buccal basal bone surface and lingual basal bone surface. The basal line (line L3) was marked by bisecting the Lg and Lh. Line L3 indicated the angulation of the basal bone on the selected measurement plane (Fig. 2c).

### Angle measurement

The upper internal angle (∠a) was formed by the long axis of the first molar (L1) and that of the long axis of the alveolar bone (L2). The angle a would be positive (+) when L1 was buccal to L2 above the intersection; and it would be negative (−) when L1 was lingual to L2 (Fig. 2b). The upper internal angle (∠b) was formed by the long axis of the alveolar bone (L2) and that of the long axis of the basal bone (L3). The angle b would be positive (+) when L2 was buccal to L3 above the intersection; and it would be negative (−) when L2 was lingual to L3 (Fig. 2c).

### Length measurement

The width GH was between the buccal and lingual alveolar plates at the lowest point of the alveolar bone and perpendicular to L2 (Fig. 2b). The width IJ was between the buccal and lingual basal plates at the highest point of the basal bone and perpendicular to L3 (Fig. 2c). Distance EF was between the buccal and lingual alveolar crest (Fig. 2b). The distance KL was between the midpoint of EF and GH (Fig. 2b).

### Statistical analysis

The minimum sample size of 385 subjects for the study was determined using the cross-sectional studies with 4Z_α_^2^P (1 − P) ÷ W^2^ where W is the width of confidence intervals was 1%, Z_α_ at 95% confidence interval = 1.96 [23]. The final sample size was 402 from 201patients.

All morphologic assessment and measurements were conducted by one examiner. The intra-examiner agreement was determined by comparing two repeated measurements at three randomly chosen sites taken 1 week apart using Pearson correlation. The intra-examiner agreement was 0.9998. All measurements were repeated for three times, and the mean value was used. The Cronbach’s alpha was applied to estimate the scale reliability, and the Cronbach’s alpha was 0.819, demonstrating a satisfactory internal consistency. The paired t-test and chi-square test were used to test the statistical significance. A *P* value of ≤ 0.05 was considered statistically significant. The SPSS 17.0 software (SPSS, Chicago, IL) was used for the statistical analysis.

## Results

### Baseline data

A total of 201 patients, consisting of 100 male and 101 female, aged between 18 and 66, who were admitted to our hospital from June 2018 to April 2020 were recruited in this investigation.

### ∠a (36), ∠a (46), ∠b (36) and ∠b (46) analysis

The ∠a (36), ∠a (46), ∠b (36) and ∠b (46) followed the normal distribution. The ∠a (36), ∠a (46), ∠b (36), and ∠b (46) were 1.97 ± 6.19, 3.12 ± 5.82, 4.40 ± 8.31, and 2.96 ± 8.85, respectively. No statistical significance was observed between the ∠a (36) and ∠a (46) group, and between the ∠b (36) and ∠b (46) group from the paired t-test (*P* > 0.05). The ∠a (36), ∠a (46), ∠b (36) and ∠b (46) for the females were 0.95 ± 5.69, 2.29 ± 5.39, 4.00 ± 7.26 and 2.44 ± 7.88, respectively, which did not significantly differ from 2.99 ± 6.53, 3.97 ± 6.14, 4.80 ± 9.28 and 3.49 ± 9.75 in the male counterparts (all *P* > 0.05). No statistical significance was noted between ∠a (36) and ∠a (46), as well as between ∠b (36) and ∠b (46) among different age groups, from the stratified analysis based on age (Table 1).

**Table 1:** Comparison of ∠a (36), ∠a (46), ∠b (36) and ∠b (46) in different age groups

Group	∠a (36)	∠a (46)	∠b (36)	∠b (46)
Aged 18–29 (n = 64)	2.12 ± 7.28	3.67 ± 6.04	3.87 ± 8.10	1.57 ± 8.23
Aged 30–39 (n = 53)	1.92 ± 5.71	2.20 ± 5.75	4.48 ± 9.36	3.90 ± 9.63
Aged 40–49 (n = 52)	2.42 ± 5.55	3.46 ± 6.23	5.16 ± 8.09	2.64 ± 9.05
Aged 50–59 (n = 22)	1.52 ± 5.63	3.35 ± 5.02	4.26 ± 8.02	4.65 ± 7.68
Aged 60–69 (n = 10)	− 0.06 ± 6.11	2.29 ± 4.39	3.67 ± 6.58	4.92 ± 9.94

### EF, GH and IJ measurement

The EF, GH and IJ were (10.83 ± 1.31) mm, (13.93 ± 2.00) mm, and (12.68 ± 1.96) mm at the left lower first molar respectively; and (10.87 ± 1.24) mm, (13.86 ± 1.93) mm, and (12.60 ± 1.90) mm at the right lower first molar respectively. No statistical significance for EF, GH, IJ were observed between the left and right lower first molar, as well as for gender and age from the paired *t*-test.

### KL and P measurement

The KL was (13.43 ± 2.21) mm at the left lower first molar. The percentage was 96.5%, 76.1% and 34.8 when the KL was over 10 mm, 12 mm and 14 mm, respectively.

The KL was (13.88 ± 2.25) mm at the right lower first molar. The percentage was 97.5%, 78.1% and 48.3% when the KL was over 10 mm, 12 mm and 14 mm, respectively.

### Classification of posterior mandibular teeth

According to the cross-sectional morphology of the mandible, the posterior teeth of the mandible could be divided into three categories with two subcategories for each.

#### The straight type

**Fig. 3:**
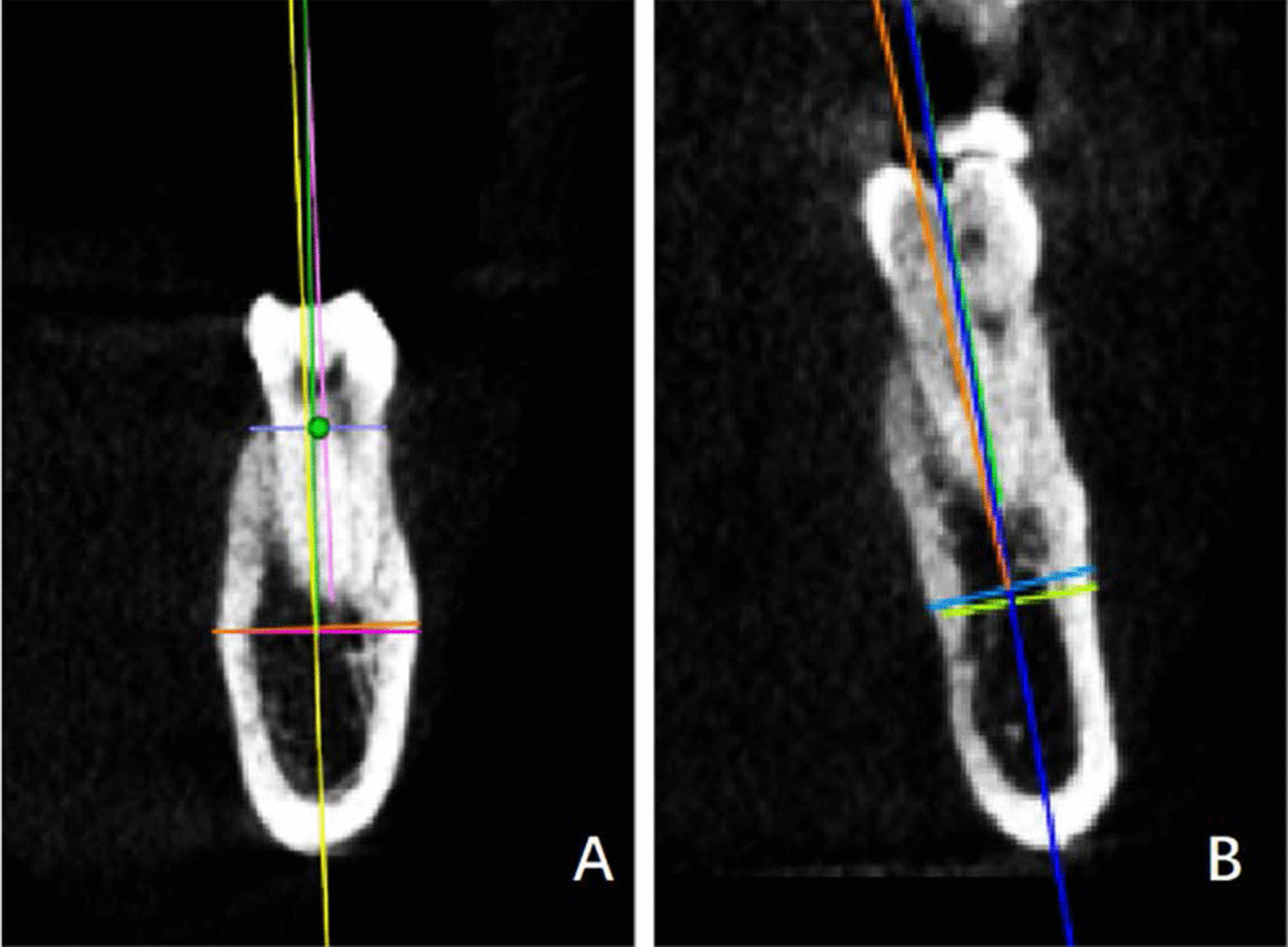
The straight type **a** I: L2 and L3 were overlapped or nearly overlapped. GH/IJ was small whereas GH was large. **b** II: L2 and L3 were overlapped or nearly overlapped. Both GH/IJ and GH were small

i. The basal bone and alveolar process were nearly aligned (L2 and L3 overlapped or nearly overlapped). GH/IJ was small whereas GH was large (Fig. 3a);
ii. The basal bone and alveolar process were nearly aligned. Both GH/IJ and GH were small (Fig. 3b). At the left lower first molar, the first subcategory of the straight type accounted for 65.7% (132/201), and merely 3.5% (7/201) for the second subcategory. At the right lower first molar, in the straight type, the first subcategory accounted for 64.2% (129/201), and the second subcategory occupied 4.5% (9/201).

#### The oblique type

**Fig. 4:**
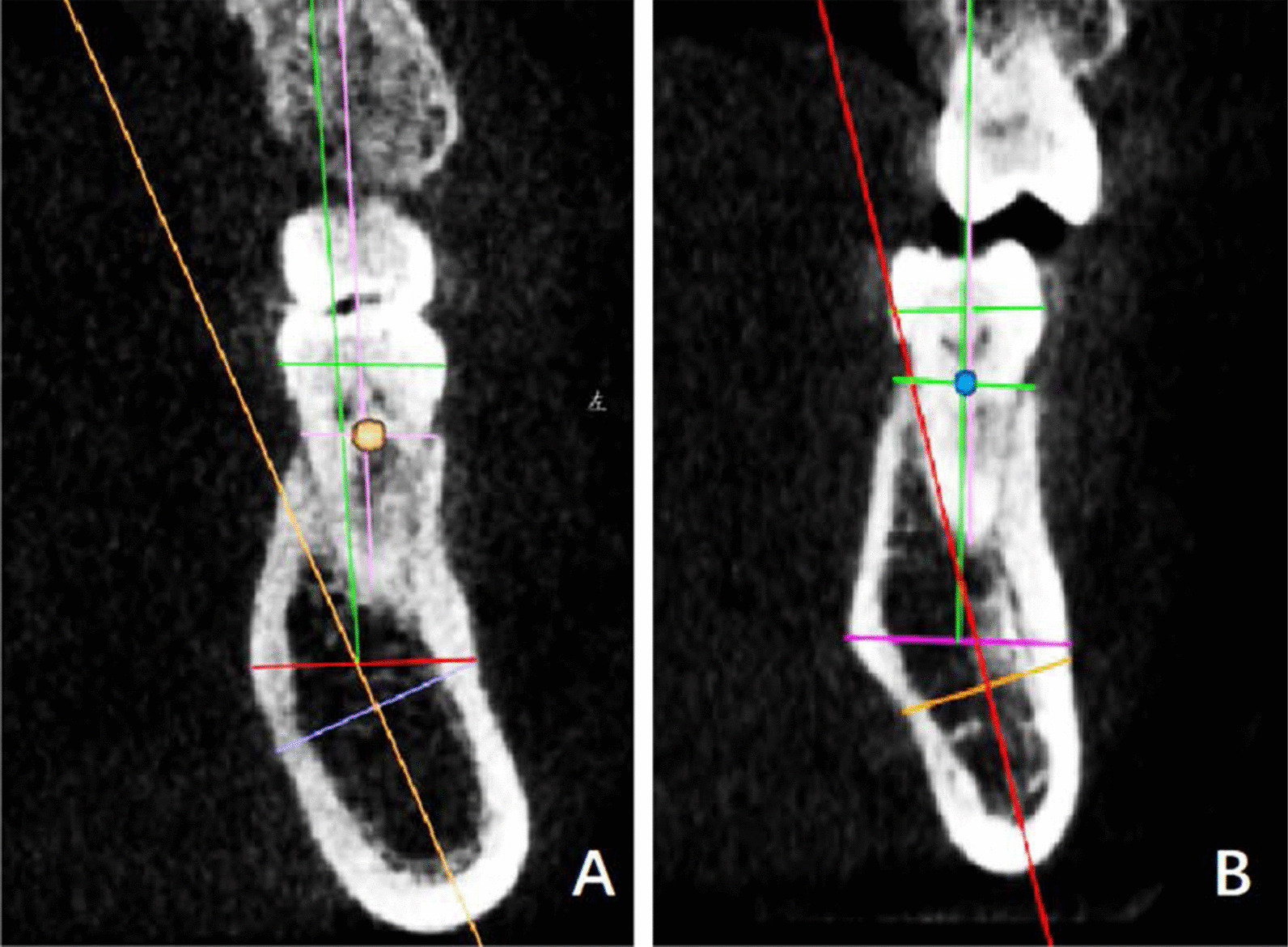
The oblique type **a** I: angle b was positively large, whereas GH/IJ was small. **b** II: angle b was positively large, whereas GH/IJ was small

i. The alveolar process was buccally angled with the basal bone (angle *b* was positively large), whereas GH/IJ was small (Fig. 4a);
ii. The alveolar process was buccally angled with the basal bone and GH/IJ was large (Fig. 4b). At the left lower first molar, the first subcategory of the oblique type occupied 10.4% (21/201), and the second subcategory accounted for 9.5% (19/201). At the right lower first molar, in the oblique type, the first subcategory was 10.0% (20/201), and the second subcategory accounted for 10.4% (21/201).

#### The concave type

**Fig. 5:**
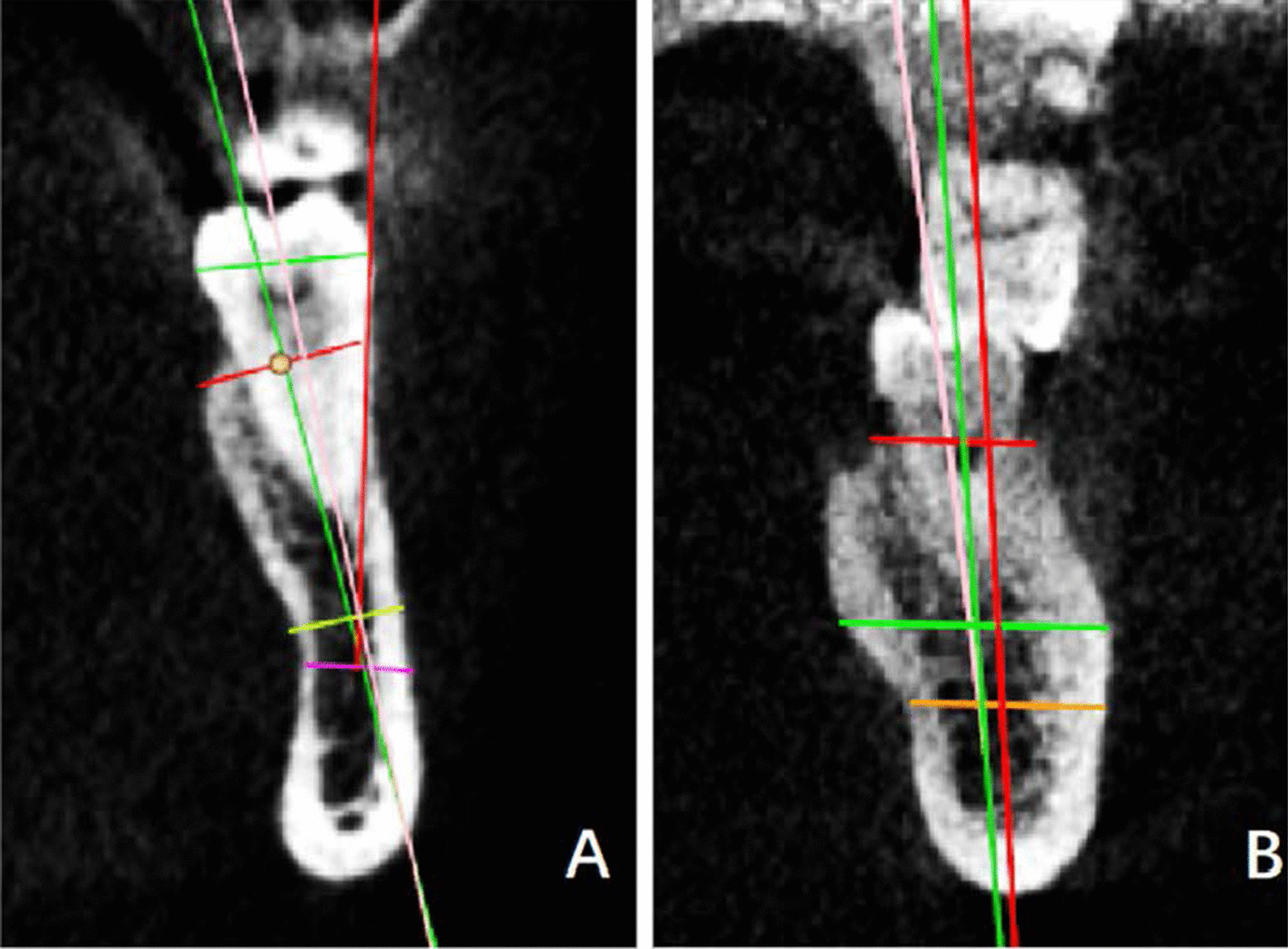
The concave type **a** I: angle b was negatively large. **b** II: L2 and L3 were nearly aligned and GH/IJ was large

i. The alveolar process was lingually angled with the basal bone (angle *b* was negatively large) (Fig. 5a);
ii. The basal bone and alveolar process were nearly aligned and GH/IJ was large (Fig. 5b). At the left lower first molar, in the concave type, the first subcategory accounted for 3.0% (6/201), and the second subcategory was 8.0% (16/201). At the right lower first molar, in the concave type, the first subcategory accounted for 3.5% (7/201), and the second subcategory was 7.5% (15/201).

**Fig. 6:**
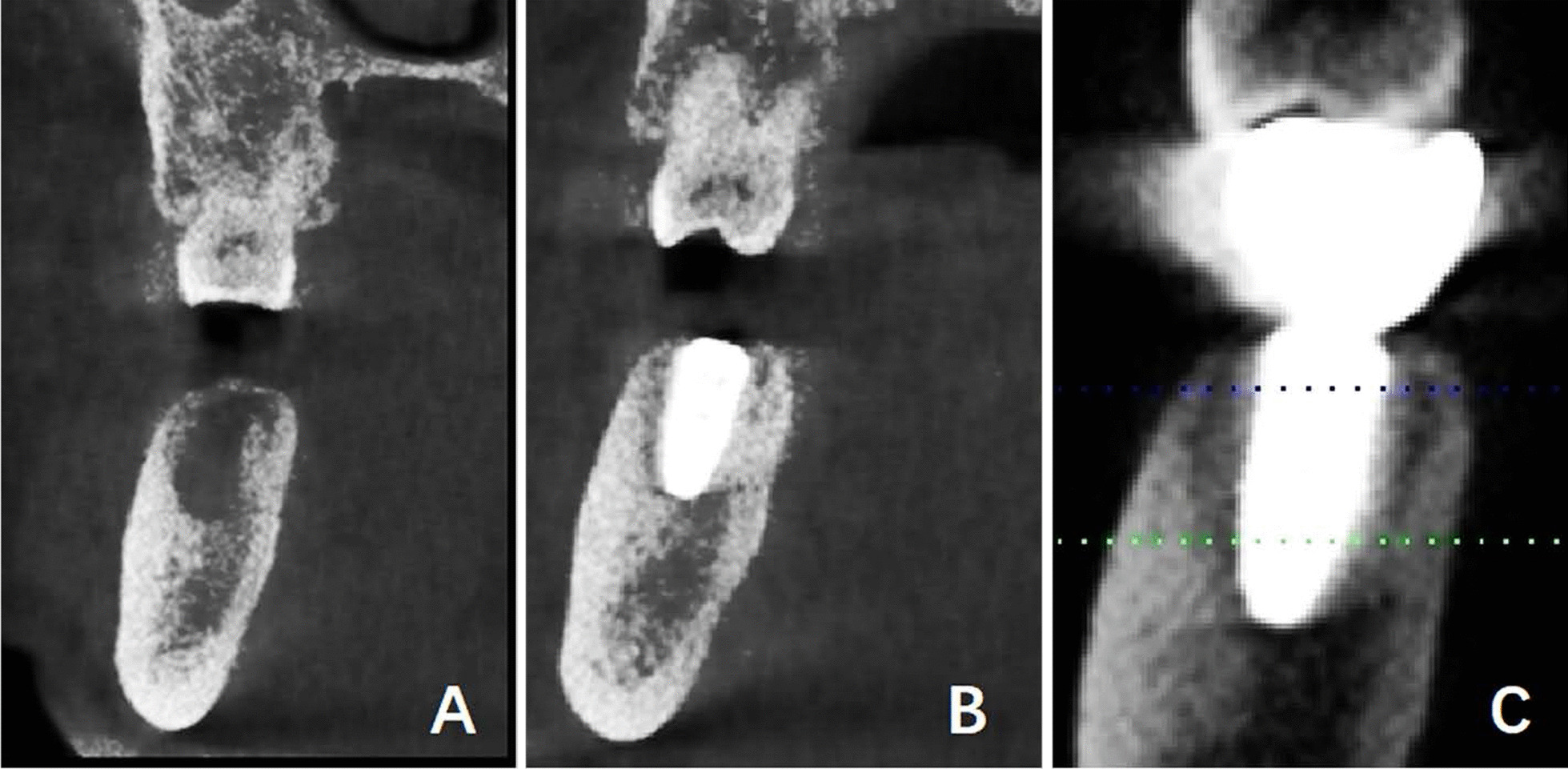
The placement of an implant in the straight type of cross-sectional posterior mandibular morphology. **a** The first subcategory of the straight type. **b** Cross-sectional CBCT image demonstrating dental implant insertion in the alveolar bone at the first molars. **c** Cross-sectional CBCT image after the prosthetic rehabilitation

**Fig. 7:**
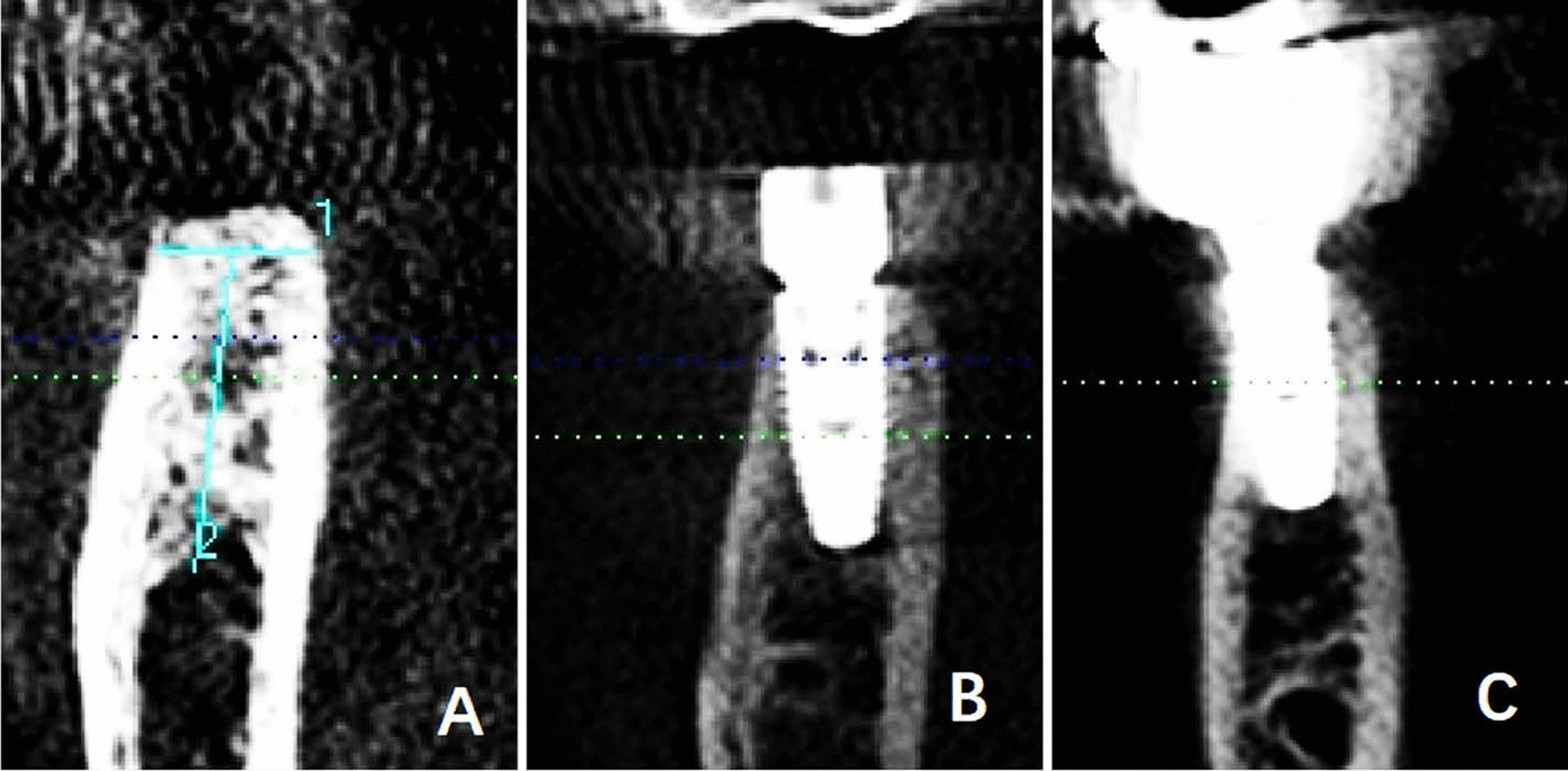
The placement of an implant in the straight type of cross-sectional posterior mandibular morphology. **a** The second subcategory of the straight type. **b** Cross-sectional CBCT image demonstrating dental implant insertion in the alveolar bone at the first molars. **c** Cross-sectional CBCT image after the prosthetic rehabilitation. A proper implant diameter should be decided based on the width of alveolar bone

The straight type was the most widely distributed, roughly accounting for 68.7–69.2%. Figure 6 demonstrated the placement of an implant in the first subcategory of the straight type. In Fig. 7, a proper implant was used for the second subcategory of the straight type, accounting for 3.5–4.5%.

**Fig. 8:**
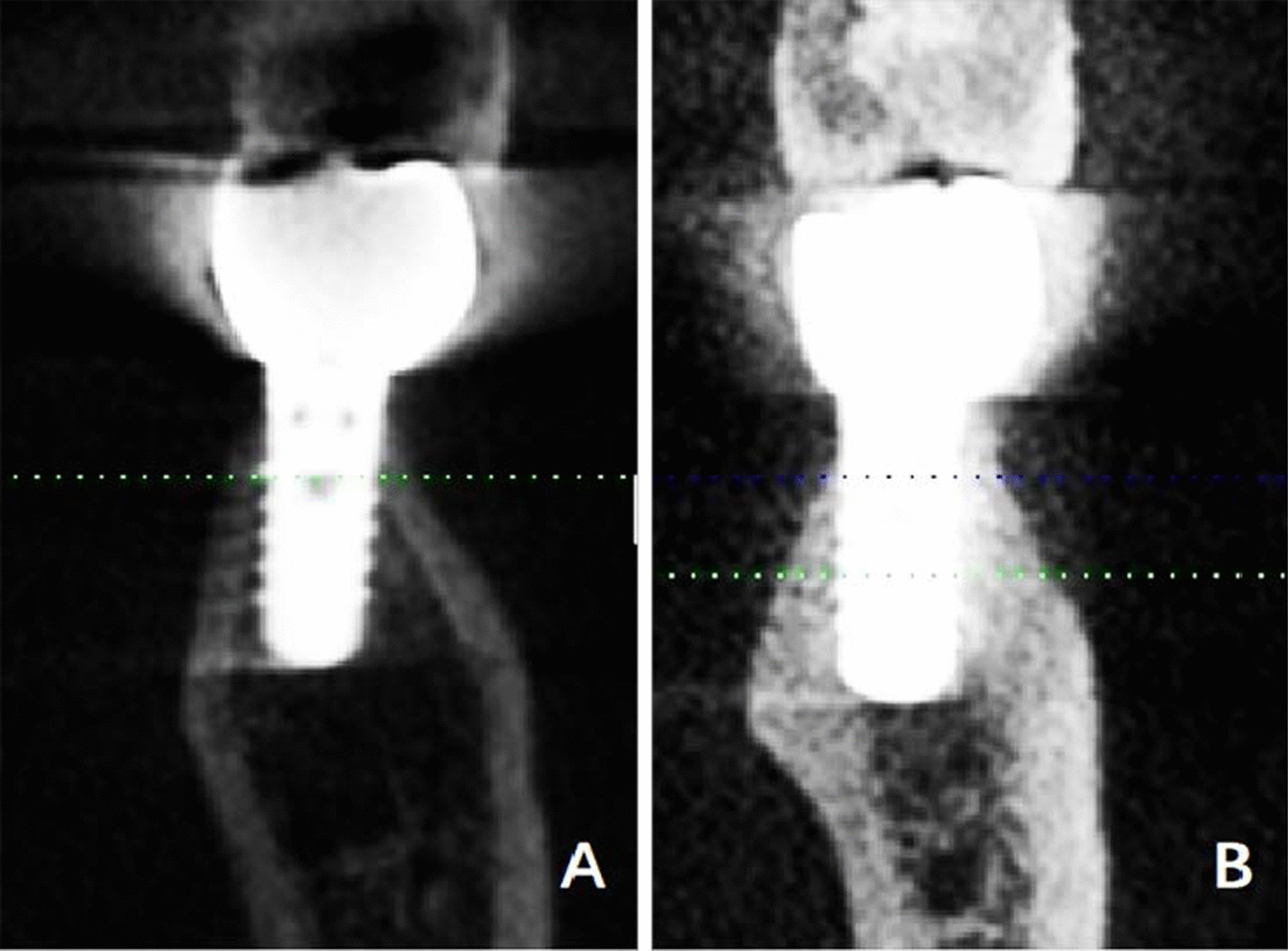
Cross-sectional views showed the buccolingual orientation of implants in the oblique type. **a** The first subcategory of the concave type after the prosthetic rehabilitation. **b** The second subcategory of the concave type after the prosthetic rehabilitation. A shorter tapered implant can be selected

The oblique type accounted for 19.9–20.4% of the mandibular first molar. Figure 8 demonstrated the placement of implants in the oblique type.

**Fig. 9:**
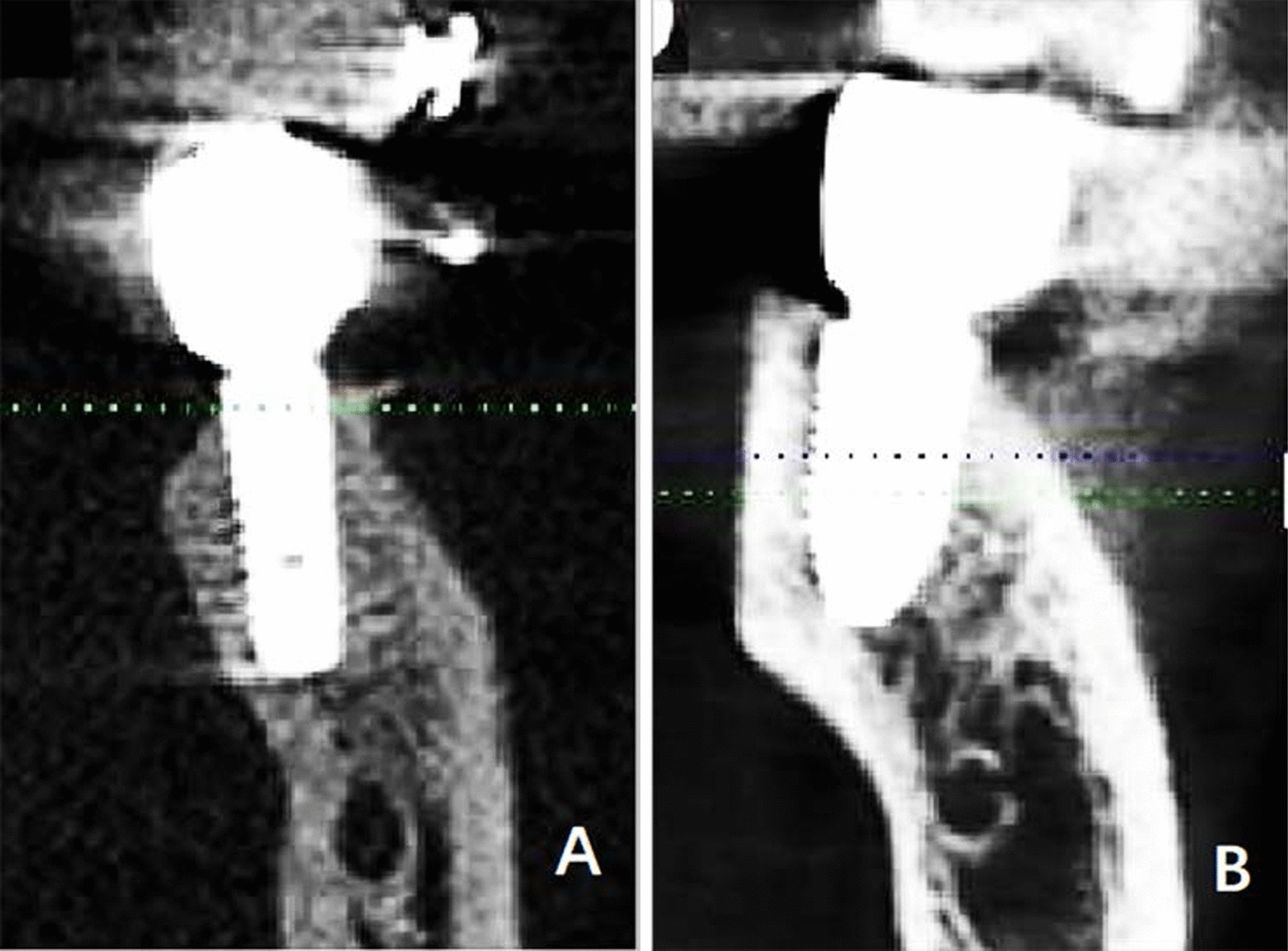
Cross-sectional views showed the buccolingual orientation of implants in the oblique type. **a** The first subcategory of the concave type after the prosthetic rehabilitation. **b** The second subcategory of the concave type after the prosthetic rehabilitation. Pay attention to the implant length, width and embedded direction to avoid lingual perforation

The concave type accounted for 11%. Figure 9 demonstrated the placement of implants in the concave type.

## Discussion

An oral surgeon is required to fully understand the morphology of the planting area to prevent complications [1, 4, 5]. The mandibular posterior lingual concavity is a common finding. The deeper the depression, the higher the risk of perforation [4, 18]. In recent years, issues of dental implantation in the posterior mandibular region have been reported, such as the plunging ranula, bleeding and pain in the sublingual area [4, 6]. If the perforation is above the mylohyoid ridge, the lingual nerve might be injured [14]. The violation of the lingual plate in the posterior mandible does not immediately result in massive bleeding and nerve injury. However, the extruded implant may be a source of persistent inflammation or infection. If left unattended, the infection might spread to the parapharyngeal and retropharyngeal space, leading to more severe complications [4, 14, 24–27].

Authors like Quirynen et al., Watanabe et al., Herranz-Aparicio et al., and Parnia et al. had adopted CT to measure and classify the mandibular morphology [4, 10, 13, 18]. Compared to panoramic radiography with low accuracy, high cost, high-dose radiation and limitation to only two-dimensional CT, the CBCT can fully evaluate the height, width, density, morphology and adjacent anatomical structures in the implanted area with a lower dose radiation [15–17]. Chan et al. and Gallucci et al. utilized CBCT [1, 9]. Quirynen et al. measured the lingual depression in the interforaminal region. The detection frequency of type I (lingual concave morphology) and type II (lingual slope morphology) was significantly lower than for type III (no concavity) [10]. Gallucci et al. measured that the frequency of s-shape and hourglass shape was significantly lower than other types [9]. In the present study, the percentages were in line with Gallucci et al. and Watanabe et al. Based on the outlines of the lingual and buccal plates, Watanabe et al. classified posterior mandibular cross-sectional morphology into types A, B and C. Type C (round) was the most commonly find, followed by type A (lingual concavity) [13]. Chan et al. calculated the proportion of mandibular lingual concavities in edentulous first molar regions reported the marked lingual undercut (U type) to be the most common type [1, 14]. Their reported prevalence of the lingual concavity was higher than that in the present article. The discrepancy between previous findings and the present study may be due to varying classification methods, the difference in cross-sectional selection, the absence of teeth and ethnic differences.

Now a days, implant treatment adopts the "crown-down" approach to focus on aesthetic restoration and long-term stability [1, 2, 6]. The long axis of occlusal forces of the implant should be as consistent as the original long axis of occlusal force of the missing teeth, because the jawbone can resist more compressive force than the tensile and shear stress [1, 5, 6]. The design idea and classification of this study was from the perspective of occlusal forces. The included angle between the tooth axis of the mandibular first molar and the alveolar bone axis was small, and independent of gender and age. No significant difference was observed in the included angles of the tooth axis, the alveolar bone axis and the basal bone axis on bilateral sides. Hence, the implant direction was generally adjacent to the alveolar bone axis. The implant scheme could be determined by referring to the direction of the tooth axis and the jaw shape of the homonymous teeth on the opposite side. The comprehensive analysis about morphological classification could be used to provide additional guidance for implant treatments. In the present study, the cross-sectional views of the posterior mandible yielded three well-differentiated morphologies based on aesthetic restoration and long-term stability. In the straight type, the basal bone and alveolar process were nearly aligned. In the oblique type, the alveolar process was buccally angled with respect to the basal bone. In the concave type, there was a marked lingual concavity. Among them, the straight type was the most widely distributed. The detection frequency of the oblique type and the concave type was significantly lower than for the straight type. The straight type was the most widely distributed, which was the most suitable for dental implanting and could be planted along the long axis of occlusal force. However, a proper implant diameter should be decided based on the width of alveolar bone for the second subcategory of the straight type. In the oblique type, the implant length and direction should be considered during surgery in consideration of the existence of the angle between the basal and alveolar bones. A shorter tapered implant can be selected when necessary to slightly deviate from the long axis of occlusal force to ensure that the lingual side would not be perforated. The implant of the concave type was the most difficult with the highest risk of lingual perforation. Extensive attention should be paid to the implant length, width and embedded direction. The tapered implant with a narrow diameter and shorter length should be used along the long axis of the alveolar bone implant. The upper repair was performed using the angle base stations to avoid lingual perforation [6, 27, 28]. When a significant lingual concavity is encountered, a CBCT scan with a radiographic guide may be indicated preoperatively so that the implant angulation in relation to this anatomic limitation can be assessed.

But alveolar bone atrophy after tooth loss did not be taken into account in this classification. As we know, tooth loss leads invariably to progressive irreversible alveolar bone atrophy [29]. Future research should focus on alveolar bone atrophy, which may refine the classification of the posterior mandibular morphology.

## Conclusion

The morphology influenced the ease or difficulty of placing an implant. This study found that implant direction was generally adjacent to the alveolar bone axis and the implant scheme could be determined by referring to the direction of the tooth axis and the jaw shape of the homonymous teeth on the opposite side in the mandibular first molar. This study based upon CBCT observations, identified three types (straight, oblique and concave types) in the mandibular first molar which can provide evidence for appropriate selection and direction design of the mandibular molar implant for dentists in clinical practice. The straight type was the most suitable for dental implanting. In the oblique type (19.9–20.4%), the implant length and direction should be considered. The concave type (11%) was the most difficult to implant with the highest risk of lingual perforation and the implant length, width, direction required more attention.

## Data Availability

The data used and/or analyzed during the current study are available from the corresponding author on reasonable request.

## Abbreviations

CT: Computed tomography
CBCT: Cone-beam CT
